# Interaction of miR-181b and *IFNA1* Polymorphisms on the Risk of Systemic Lupus Erythematosus

**DOI:** 10.1155/2020/4757065

**Published:** 2020-04-23

**Authors:** Yong-Ling He, Jun Yang, Zhi-Neng Zeng, Xiang Shi

**Affiliations:** Department of Laboratory Medicine, Affiliated Hospital of Guilin Medical University, Guilin, Guangxi, China

## Abstract

**Introduction:**

A previous work has discovered that chromosome 1q32 locus linked to the risk of systemic lupus erythematosus (SLE) and miR-181b located on the susceptibility site with downregulation inversely correlating to its target molecular interferon alpha 1 (*IFNA1*). The purpose of this study was to investigate the association of miR-181b and *IFNA1* polymorphisms with IS risk.

**Methods:**

The miR-181b rs322931, *IFNA1* rs1332190, and rs10811543 were genotyped using a Multiplex SNaPshot assay. miR-181b expression levels in plasma of SLE patients and controls were analyzed using quantitative PCR.

**Results:**

The rs322931 CT, CT/TT, and T allele exerted an increased trend of SLE risk (CT vs. CC: adjusted OR = 1.71, 95% CI 1.16-2.50, *P* = 0.01; CT/TT vs. CC: adjusted OR = 1.45, 95% CI 1.08-1.95, *P* = 0.01; T vs. C: adjusted OR = 1.38, 95% CI 1.07-1.79, *P* = 0.01). Combined genotypes of the rs322931 CT/TT+rs1332190 TT and the rs322931 CC+rs10811543 AG/AA also revealed an increased risk of SLE. Gene-gene interaction analysis showed that a three-locus model consisting of rs322931, rs1332190, and rs10811543 attributed an increased risk of SLE. Further genotype-phenotype analysis revealed that rs322931 CT/TT carriers displayed lower levels of miR-181b.

**Conclusions:**

These findings indicate that the miR-181b rs322931 may be singly and jointly responsible for the etiology of SLE by altering miR-181b expression.

## 1. Introduction

Systemic lupus erythematosus (SLE), a chronic inflammatory disease characterized by multiple immunologic abnormalities, can damage many organs [1]. The prevalence rates of SLE are about 17-48/100,000 population worldwide, and females are 3-6 times more frequently affected than males. The burden of SLE is not only physical and mental health but also socioeconomic impact because the most common age of onset is 20-40 years, and patients with that age are still raising or supporting families [2]. Risk factors of SLE included cigarette smoking, oxidative stress, ultraviolet light, infection, and hormonal action as well as genetic factors [3–12]. Environmental exposure may trigger SLE in individuals who carry a predisposing background of genetic susceptibility [2, 4, 7]. Several single-nucleotide polymorphisms (SNPs) in coding genes have been found to be involved in the pathogenesis of SLE, such as rs1051169 in *S100B* [8]; rs20541 in interleukin- (*IL*-) 13 [9]; rs11556218, rs4778889, and rs4072111 in *IL-16* [10]; rs2227513 in *IL-22* [11]; and rs7977932 in *IL-31* [12].

In addition to coding genes, noncoding transcripts, such as microRNAs (miRNAs) also play a critical role in modulating immune response of SLE [13–16]. Until today, more than 1500 miRNAs have been sequenced in human [17], with an estimation of regulating over one-third of genome expression [18]. Among them, the miR-181 family was found to be downregulated in patients with SLE, being an important modulator of B and T cell differentiation and inflammatory reaction, which were key events in the initiation and development of SLE [13, 19–22].

Recently, miRNA-related genetic variant has been reported to be a risk factor for a series of human diseases, including SLE [23–30]. For example, an SNP rs4937333 T allele was reported to be associated with a significantly increased risk of SLE by enhancing the binding of miR-5003 to transcriptional factor *ETS1* and decreasing *ETS1* expression [26]. A genetic variant of rs322931 was reported to alter both transcriptional activity and the expression of miR-181b [29, 30]. To date, no study reported the association of miR-181b rs322931 with the risk of SLE. In this hospital-based case-control study, we investigated whether the miR-181b rs322931 was related to the occurrence of SLE in a Chinese Han population. As miR-181b may contribute to the development and progression of SLE through directly targeting molecular interferon alpha 1 (*IFNA1*) [13], two SNPs (i.e., rs1332190 and rs10811543) in the promoter region of *IFNA1* were also examined for gene-gene interaction analysis. We found that the miR-181b rs322931 and *IFNA1* rs1332190 and rs10811543 may play an interactive role in the etiology of SLE.

## 2. Materials and Methods

### 2.1. Study Population

A total of 402 SLE patients and 430 age- and sex-matched healthy controls were included in this study. Patients with SLE were consecutively enrolled from the Affiliated Hospital of Guilin Medical University between May 2012 and December 2018. All patients fulfilled the American College of Rheumatology criteria for SLE (1997) [31]. The controls were also registered from the same hospital at the same period. We excluded those controls if they had autoimmune diseases, such as malar butterfly rash, photosensitivity, fever, erythra, and edema. All the subjects without blood transfusion were genetically unrelated ethnic Han Chinese living in Guangxi province. After written informed consent was signed, each participant donated 3-5 mL of eathylene diamine tetraacetic acid-anticoagulated peripheral venous blood. Plasma was separated by centrifuging for 10 min at 1000 rpm and stored in -80°C until analysis. The study protocol was approved by the Institutional Review Board of the Affiliated Hospital of Guilin Medical University.

### 2.2. SNP Selection

We selected miR-181b rs322931 locating on the chromosome 1q32 that has been identified to be a risk locus of SLE [32]. Additionally, we selected SNPs within the *IFNA1* that is a target gene of miR-181b [32], with the inclusion criteria of minor allele frequency > 10% in Chinese Han population. Two SNPs (i.e., rs1332190 and rs10811543) in the promoter of *IFNA1* were selected.

### 2.3. Genotyping

Genomic DNA was extracted from leukocytes of peripheral blood using the commercially available kit (Tiangen, Beijing, China). The miR-181b rs322931 and *IFNA1* rs1332190 and rs10811543 were genotyped using a Multiplex SNaPshot assay. For quality control, negative control replacing template DNA with distilled water was used in each run and about 5% of all samples were randomly selected for validation using DNA sequencing. The results between the two genotyping methods were concordant.

### 2.4. Quantitative PCR (qPCR) of miR-181b

Total RNA was isolated from plasma using a commercial kit (Qiagen, Hilden, Germany) following the manufacturer's protocol. cDNA was generated using the Mir-X miRNA First-Strand Synthesis Kit (Takara Bio USA, Mountain View, CA, USA) according to the manufacturer's manual. miR-181b levels in cases and controls were quantified using the Mir-X miRNA qRT-PCR TB Green Kit (Takara Bio USA). qPCR was run on an ABI 7900HT real-time PCR machine (Applied Biosystems, CA, USA). U6 was used as an internal control, and relative expression of miR-181b was determined using the 2^-*ΔΔ*Ct^ method [33].

### 2.5. Statistical Analysis

Statistical analysis was done using SPSS software version 13.0 (SPSS, Chicago, IL, USA). The *χ*^2^ test was used to evaluate Hardy-Weinberg equilibrium (HWE) and the differences of the rs322931, rs1332190, and rs10811543 between cases and controls. Odds ratios (ORs) and 95% confidence intervals (CIs) were used to estimate the association between each SNP and SLE risk using a logistic regression model after adjustment for age and gender. Linkage disequilibrium (LD) and haplotype analysis were performed using the SHEsis software [34]. The statistical significant value of SNPs was set as 5 × 10^−8^ that was used in genome-wide association study. Gene-gene interaction analysis was performed by using multifactor dimensionality reduction (MDR) platform [35]. miR-181b expression levels were compared using the Mann–Whitney *U* test, and a value of *P* < 0.05 was considered statistically significant.

## 3. Results

### 3.1. Characteristics of Study Population

The characteristics of the study population are summarized in Table 1. There were no significant differences in the distribution of age and gender (*P* = 0.27 and 0.10, respectively). Approximately half of the SLE patients had photosensitivity, leucopenia, anaemia, complement depression, renal disorder, and arthritis, while only about 30% patients had malar rash and negative AdsA. Most of the patients (87.3%) had positive ANA.

### 3.2. Main Effects of SNPs in miR-181b and *IFNA1* on SLE Risk

The genotype and allelic frequencies of the selected SNPs among cases and controls are presented in Table 2. The genotype distributions of the miR-181b rs322931 and *IFNA1* rs1332190 and rs10811543 met the HWE requirements in both cases and controls (*P* > 0.05). Compared to the miR-181b rs322931 CC genotype, individuals with the CT variant genotype exerted an increased trend of SLE risk (adjusted OR = 1.71, 95% CI 1.16-2.50, *P* = 0.01). Under the dominant genetic of inheritance, carriers with the CT/TT genotypes had a 1.45-fold increased risk of SLE (adjusted OR = 1.45, 95% CI 1.08-1.95, *P* = 0.01). Moreover, carriers with the T allele had a 1.38-fold increased risk of SLE (adjusted OR = 1.38, 95% CI 1.07-1.79, *P* = 0.01). For the rs1332190 and rs10811543, no significant difference between SLE patients and controls was found (*P* > 0.05). Stratification analysis also showed null association between the three SNPs and clinical features of SLE (Table 3).

### 3.3. Haplotype and Combined Analysis

LD results revealed that the rs1332190 and rs10811543 were in strong LD (D′ = 0.97, *r*^2^ = 0.67). The frequencies of 4 possible haplotypes are shown in Table 4. There was no significant difference of the haplotype between cases and controls.

Compared to the combined genotypes rs322931 CC+rs1332190 TT, the combined genotypes rs322931 CT/TT+rs1332190 TT and rs322931 CC+rs1332190 CT/CC were associated with a reduced risk of SLE (OR = 0.06, 95% CI 0.02-0.18, *P* = 2.55 × 10^−11^; adjusted OR = 0.37, 95% CI 0.26-0.53, *P* = 3.74 × 10^−8^, respectively). Compared to the combined genotypes rs322931 CC+rs10811543 GG, the combined genotypes rs322931 CC+rs10811543 AG/AA were associated with a reduced risk of SLE (OR = 0.33, 95% CI 0.22-0.49, *P* = 3.07 × 10^−8^) (Table 5).

### 3.4. Interaction Analysis

Table 6 shows interaction analysis among the miR-181b rs322931 and *IFNA1* rs1332190 and rs10811543. When taking the three SNPs together, the accuracy was the highest with the cross-validation consistency of 10/10 (OR = 9.99, 95% CI 6.54-15.26, *P* < 0.001), indicating that the miR-181b rs322931 and *IFNA1* rs1332190 and rs10811543 were interactively associated with the risk of SLE.

### 3.5. The rs322931 CT/TT Exhibited Lower Levels of miR-181b

**Figure 1:**
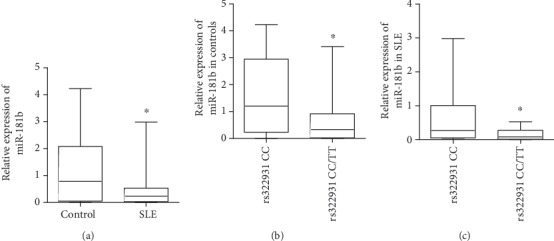
The rs322931 CT/TT exhibited lower levels of miR-181b. (a) Relative expression levels of miR-181b in controls (*n* = 44) and SLE patients (*n* = 42). (b, c) The association of the rs322931 with miR-181b expression in controls and SLE patients (^∗^*P* < 0.05).

The expression levels of miR-181b were analyzed using qPCR in controls (*n* = 44) and SLE patients (*n* = 42). As shown in Figure 1(a), the miR-181b expression was lower in SLE patients compared to controls (*P* < 0.05). When comparing the relationship of the rs322931 to miR-181b expression, we found that the rs322931 CT/TT carriers had lower levels of miR-181b than rs322931 CC carriers (*P* < 0.05) (Figures 1(b) and 1(c)).

## 4. Discussion

In this hospital-based case-control study, we found that the miR-181b rs322931 C but not *IFNA1* rs1332190 and rs10811543 was associated with an increased trend of SLE risk, whereas combined analysis showed a significant association of rs322931-rs1332190 and rs322931-rs10811543 with SLE risk. Gene-gene interaction analysis also showed that the three-locus model consisting of rs322931, rs1332190, and rs10811543 attributed an increased risk of SLE. In addition, genotype-phenotype analysis revealed that the rs322931 CT/TT carriers displayed lower levels of miR-181b. Our data shed light on the importance of miR-181b rs322931 in the setting of SLE.

The miR-181 family including miR-181a, miR-181b, miR-181c, and miR-181d is a critical regulator in the pathogenesis of SLE [13–16, 19, 36, 37]. Altered expression of miR-181a was not only observed in SLE and lupus nephritis patients but also correlated to clinical features, such as the erythrocyte sedimentation rate, C reactive protein, anti-dsDNA antibody, complements, and score of SLE disease activity index [14–16, 36, 37]. miR-181b was also reported to be differentially expressed in SLE patients [13]. These findings indicate that miR-181 may be a biomarker for the diagnosis of SLE and monitor of SLE activity.

Previously, an SNP rs322931 was found to be a risk factor for positive emotion, reward processing, and ischaemic stroke through influencing transcriptional activity and the expression levels of miR-181b [29, 30, 38]. The current work has discovered that chromosome 1q32 locus linked to the risk of SLE and miR-181b located on the susceptibility site with the downregulation inversely correlating to its target molecular *IFNA1* [32]. Based on this background, we hypothesized that the rs322931 may affect individual's susceptibility to SLE. Our findings in this association study confirmed this hypothesis. We found that the rs322931 CT/TT genotypes and T allele carriers had a 1.45-fold and 1.38-fold increased risk of SLE, respectively. qPCR was then performed to detect the expression of miR-181b, and we found that the miR-181b levels were significantly lower in SLE patients than controls, confirming previous report [32]. Of note, after comparing the correlation of the rs322931 to miR-181b expression, we found that carriers with the rs322931 CT/TT genotypes exhibited lower levels of miR-181b in both SLE patients and controls. Taken together, we may conclude that the rs322931 CT/TT genotypes and T allele contributed to the risk of SLE by decreasing the levels of miR-181b.

In addition to the miR-181b rs322931, we examined much more SNPs in this study because SLE is a complex disease and involved in more than one gene. Genome-wide association study has provided evidence of susceptibility loci of SLE in the interferon (IFN) signaling pathway [39]. IFN-*α* is highly expressed and has emerged as a key pathogenic cytokine in SLE [40, 41]. For example, IFN-*α* in sera from active SLE patients can induce differentiation of dendritic cells that capture and present antigens to CD4+ T cells [42]. IFN-*α* also exerts stimulatory effects on the adaptive immune system by enhancing B cell differentiation and survival of autoimmune B cells [43, 44]. Moreover, IFN-*α* impairs autophagic degradation of mtDNA and vasculogenesis in SLE, serving as a drug target [45, 46]. IFN-*α* is encoded by *IFNA1* that is a target gene of miR-181b [13]. Genetic polymorphisms in *IFNA1*, therefore, were analyzed in this study. Although no significant association of the rs1332190 and rs10811543 in the promoter of *IFNA1* with SLE risk was observed in single site analysis, combined analysis revealed an association of the 2 SNPs with SLE occurrence. Both the rs322931 CT/TT+rs1332190 CT/CC and the rs322931 CC+rs10811543 AG/AA were associated with a reduced risk of SLE. Finally, we performed miR-181b/*IFNA1* interaction analysis, and we found that the miR-181b rs322931/rs1332190/rs10811543 was the best candidate model with the accuracy of 0.67. Our findings indicate that the miR-181b rs322931 may be singly and jointly responsible for the etiology of SLE.

Some limitations should be discussed in the interpretation of our data. It is evident that nongenetic factors such as cigarette smoking, oxidative stress, ultraviolet light, infection, and hormonal action play important roles in the development of SLE [3–6]. In this study, we did not collect these factors and thus, the association of miR-181b and *IFNA1* polymorphisms with environment factors cannot be analyzed. Additionally, the samples in this study were not large enough and all of them were enrolled from Han Chinese. Further studies with larger sample sizes are required to assess whether our positive findings can be confirmed in other ethnicities.

In summary, we found for the first time that the rs322931 C risk allele was related to the development of SLE and the rs322931 CT/TT genotypes altered miR-181b expression levels in SLE patients. Of note, miR-181b-*IFNA1* interaction conferred the risk of SLE. Once confirmed in other ethnicities with larger sample sizes, it is likely to be important for future personalized treatment of SLE by genotyping the miR-181b rs322931 and *IFNA1* rs1332190 and rs10811543.

## Figures and Tables

**Table 1:** Characteristics of the study population.

Variables	Controls, *n* = 430	Patients with SLE, *n* = 402	*P* value
Age (mean ± SD)	34.3 (±12.2)	33.2 (±16.0)	0.27
Male/female (%)	105 (24.4)/325 (75.6)	79 (19.7)/323 (80.3)	0.10
Malar rash (%)		123 (30.6)	
Photosensitivity (%)		241 (60.0)	
Leucopenia (%)		214 (53.2)	
Anaemia (%)		224 (55.7)	
Complement depression (%)		264 (65.8)	
Renal disorder (%)		205 (51.0)	
Arthritis (%)		235 (58.5)	
ANA (%)		351 (87.3)	
AdsA (%)		272 (67.7)	

SLE: systemic lupus erythematosus; SD: standard deviation; ANA: antinuclear antibodies; AdsA: anti-double stranded DNA antibody.

**Table 2:** Association between SNPs in miR-181b and *IFNA1* and risk of SLE.

Polymorphisms	Controls, *n* = 430 (%)	SLE, *n* = 402 (%)	Adjusted OR (95% CI)^†^	*P* value
miR-181b rs322931				
CC	310 (72.1)	257 (63.9)	1.00	
CT	114 (26.5)	135 (33.6)	1.71 (1.16-2.50)	0.01
CT/TT	120 (27.9)	145 (36.1)	1.45 (1.08-1.95)	0.01
C allele	734 (85.3)	649 (80.7)	1.00	
T allele	126 (14.7)	155 (19.3)	1.38 (1.07-1.79)	0.01
*IFNA1* rs1332190				
TT	220 (51.2)	199 (49.5)	1.00	
CT	185 (43.0)	168 (41.8)	1.02 (0.77-1.36)	0.89
CT/CC	210 (48.8)	203 (50.5)	1.08 (0.83-1.42)	0.56
T allele	625 (72.7)	566 (70.4)	1.00	
C allele	235 (27.3)	238 (29.6)	1.13 (0.91-1.40)	0.27
*IFNA1* rs10811543				
GG	260 (60.5)	250 (62.2)	1.00	
AG	146 (34.0)	132 (32.8)	0.96 (0.71-1.28)	0.76
AG/AA	170 (39.5)	152 (37.8)	0.95 (0.71-1.25)	0.70
G allele	666 (77.4)	632 (78.6)	1.00	
A allele	194 (22.6)	172 (21.4)	0.95 (0.75-1.20)	0.66

SNP: single-nucleotide polymorphism; *IFNA1*: interferon alpha 1; SLE: systemic lupus erythematosus; OR: odds ratio; CI: confidence interval. ^†^Adjusted by age and gender.

**Table 3:** Stratification analyses of SNPs in miR-181b and *IFNA1* and clinical features of SLE.

Variables	miR-181b rs322931 CT/TT vs. CC		*IFNA1* rs1332190 CT/CC vs. TT		*IFNA1* rs10811543 AG/AA vs. GG	
	Adjusted OR^†^	*P* value	Adjusted OR^†^	*P* value	Adjusted OR^†^	*P* value
Malar rash	1.18 (0.75-1.85)	0.47	1.09 (0.71-1.67)	0.70	1.29 (0.82-2.02)	0.26
Photosensitivity	1.18 (0.75-1.85)	0.20	0.61 (0.40-0.91)	0.02	0.78 (0.52-1.19)	0.25
Leucopenia	0.80 (0.53-1.21)	0.30	0.75 (0.51-1.12)	0.16	0.88 (0.59-1.32)	0.54
Anaemia	0.99 (0.66-1.49)	0.96	1.17 (0.79-1.74)	0.43	1.07 (0.71-1.60)	0.76
Complement depression	1.10 (0.71-1.68)	0.67	1.05 (0.69-1.58)	0.83	1.03 (0.67-1.58)	0.89
Renal disorder	1.13 (0.75-1.71)	0.55	1.36 (0.92-2.01)	0.13	1.13 (0.75-1.69)	0.56
Arthritis	0.75 (0.50-1.15)	0.19	0.92 (0.62-1.37)	0.68	1.06 (0.70-1.59)	0.80
ANA	0.87 (0.47-1.62)	0.66	0.85 (0.47-1.52)	0.58	0.71 (0.38-1.34)	0.28
AdsA	1.10 (0.71-1.70)	0.67	1.34 (0.88-2.04)	0.18	1.10 (0.72-1.70)	0.66

SNP: single-nucleotide polymorphism; *IFNA1*: interferon alpha 1; SLE: systemic lupus erythematosus; OR: odds ratio; CI: confidence interval. ^†^Adjusted by age and gender.

**Table 4:** Haplotype analysis of SNPs in *IFNA1* between cases and controls.

rs1332190	rs10811543	Controls, *n* (%)	SLE, *n* (%)	OR (95% CI)	*P* value
T	G	619 (72.0)	566 (70.4)	1.00	
C	A	188 (21.9)	172 (21.4)	1.00 (0.79-1.27)	1.00
C	G	47 (5.5)	66 (8.2)	1.54 (1.04-2.27)	0.03
T	A	6 (0.7)	0 (0.0)	—	—

SNP: single-nucleotide polymorphism; *IFNA1*: interferon alpha 1; SLE: systemic lupus erythematosus; OR: odds ratio; CI: confidence interval.

**Table 5:** Combined analyses of SNPs in miR-181b and *IFNA1* and risk of SLE.

Combined genotypes	Controls, *n* = 430 (%)	SLE, *n* = 402 (%)	OR (95% CI)	*P* value
miR-181b rs322931-*IFNA1* rs1332190				
rs322931 CC+rs1332190 TT	166 (38.6)	195 (48.5)	1.00	
rs322931 CT/TT+rs1332190 TT	54 (12.6)	4 (1.0)	0.06 (0.02-0.18)	2.55 × 10^−11^
rs322931 CC+rs1332190 CT/CC	144 (33.5)	62 (15.4)	0.37 (0.26-0.53)	3.74 × 10^−8^
rs322931 CT/TT+rs1332190 CT/CC	66 (15.3)	141 (35.1)	1.82 (1.27-2.60)	0.001
miR-181b rs322931-*IFNA1* rs10811543				
rs322931 CC+rs10811543 GG	196 (45.6)	216 (53.7)	1.00	
rs322931 CT/TT+rs10811543 GG	64 (14.9)	34 (8.5)	0.48 (0.31-0.76)	0.002
rs322931 CC+rs10811543 AG/AA	114 (26.5)	41 (10.2)	0.33 (0.22-0.49)	3.07 × 10^−8^
rs322931 CT/TT+rs10811543 AG/AA	56 (13.0)	111 (27.6)	1.80 (1.24-2.62)	0.002

SNP: single-nucleotide polymorphism; *IFNA1*: interferon alpha 1; SLE: systemic lupus erythematosus; OR: odds ratio; CI: confidence interval.

**Table 6:** Interaction analysis of SNPs in miR-181b and *IFNA1* and risk of SLE.

Best candidate models	Accuracy	Cross-validation consistency	OR (95% CI)	*P* value
rs322931	0.55	10/10	1.46 (1.09-1.95)	0.01
rs322931/rs1332190	0.67	10/10	9.53 (6.24-14.55)	<0.001
rs322931/rs1332190/rs10811543	0.67	10/10	9.99 (6.54-15.26)	<0.001

SNP: single-nucleotide polymorphism; *IFNA1*: interferon alpha 1; SLE: systemic lupus erythematosus; OR: odds ratio; CI confidence interval.

## Data Availability

The digital data used to support the findings of this study are available from the corresponding author upon request.

## Additional Points

*Key-points*. 1. Individuals with the miR-181b rs322931 CT, CT/TT, and T allele had an increased trend of SLE risk. 2. Combined analysis showed a significant association of rs322931-rs1332190 and rs322931-rs10811543 with SLE risk. 3. Gene-gene interaction analysis showed that the three-locus model consisting of rs322931, rs1332190, and rs10811543 attributed an increased risk of SLE. 4. The rs322931 CT/TT carriers displayed lower levels of miR-181b.
